# Spiritual Development and Death Attitude in Female Patients With Type II Diabetes

**Published:** 2014

**Authors:** Masoumeh Nozari, Alireza Khalilian, Yarali Dousti

**Affiliations:** 1 Department of Psychology, Islamic Azad University, Sari Branch, Sari, Iran.; 2 Professor, Psychiatry and Behavioral Research Center, Addiction Institute AND Department of Biostatistics, Mazandaran University of Medical Science, Sari, Iran.; 3 Associate Professor, Department of Psychology, Islamic Azad University, Sari Branch, Sari, Iran

**Keywords:** Death Attitude, Spiritual Development, Type II Diabetes Mellitus

## Abstract

**Objective:** The present study aimed to investigate the differences regarding spiritual development dimensions and death attitude profiles, and also to determinate association between them, in patients suffering from type II diabetes.

**Methods:** In a cross-sectional design study, 100 female outpatients who were suffering from type II diabetes were recruited in Imam Khomeini Hospital, Sari, Iran. Data were collected through two questionnaires including the Spiritual Assessment Inventory (SAI) and the Death Attitude Profile-Revised (DAPR). Analysis of the data involved analysis of covariance (ANCOVA) with the Fisher's Least Significant Difference (LSD) as post-hoc test plus the Pearson correlation.

**Results: **There was a statistical significant difference in spiritual development dimensions and death attitude profile. The results showed that spiritual development were significantly associated with some items of death attitude profiles.

**Conclusion:** Awareness of God was suitable in diabetic patients, but the quality of relationship with God indicated spiritually immature. It is necessary to provide instruction to improve patient's death attitude and following health behavior.

## Introduction

Diabetes is the most complex chronic diseases which causes innumerable psychological complications, particularly anxiety and depression (1, 2). Behaviors related to lifestyles, such as nutrition, physical activity, smoking, and social impacts associated with income inequality and negative factors of stressful conditions at workplace (lack of job security and stability) affect diabetics (3). The study of Larranaga et al. on 65,000 diabetic patients over 24 years showed that lower socioeconomic conditions is in association with higher incidence of type II diabetes and its chronic consequences (3).

Researchers noted that there is a positive association between patients' attitude and their behavioral intent with whatever an individual believes of her/his self-care (4). Furthermore, health inner control canon and autonomous casual tendency lead to increased self-care behaviors (5). Imaging neural system in studies related to assumption of "vascular depression" mentions depression as a strong predictor factor that leads to stroke and vascular problems and some mention religiousness as a protector factor (6). People who are religious have positive social relations and participate in social services activities. They usually use well-known sources such as worship, individual activity, and belief in God during periods of stress (7). Various studies have shown that there is a significnat relationship between spirituality and life purpose, life satisfaction, mental health, more longevity, happiness, life expectancy, behavior adaptation, question solution and contradiction solution (8, 9).

Alertly awareness and adoption with life events is a factor of spirituality development. Vegan considered spiritual maturity as a fact which is consisted of rate of excitatory maturity, moral maturity and ethical behavior that includes wisdoms and compassion for others regardless sex, ethnicity, age or race (10). Hall and Edwards considered spiritual maturity as a combination of two dimensions i.e. ''awareness of God'' and "the quality relationship with God''. Increase in awareness of God helps one in a way that he/she may comprehend God in all of his/her life aspects. Quality of relationship with God includes: 1) realistic acceptance (i.e. person's ability to incorporate positive experiences and despairs in person's relation with God), 2) disappointment (indication of many demands of person from God that leads to discouragement), 3) instability (person's ability to incorporate good and evil side of him/herself and excessive halving), and 4) grandiosity (person deals with the effects of tendencies toward grandiosity on relationship with God and assumes himself/herself as better than others) (11).

Neimeyer by researching on related researches to death anxiety in various religions stated that the base of fear is different in various religions regarding level of being religious. In Jewish society, moderate religious people are worry about effect of death consequences on family, while concerns of very religious people appertain to punishment in afterlife. In Christian society, fear of unknown factors has positive correlation with extrinsic religious orientation and people who have realer religious obligation, have amended their fear. Anxiety base in Muslims society was concentrated on torture and torment of the grave (12). Belief to life after death in religious people was associated with death acceptance approach while people who has surfeit attitude on religion or those who were biased in religious show more anxiety and stress concerning death that indicates their defensive state against death to process religious subjects (13, 14).

People show different responds against death anxiety. They may tend to increase or decrease health promoter behaviors (15). As Neimeyer thinks, automatically being patient leads to make of death concerns in some people. On the other hand, if health system becomes critical, levels of death anxiety can affect personal performance such as social supports and personal sources as contradictory styles and religious beliefs (12). Mehnert and Koch found that there is an association between death acceptance and mental stresses, mental style, contradictor style and quality of life (16). 

There are insufficient studies in Iran about spiritual development in diabetics. Besides, given the fact that positive change in life meaning and attitudes improvement leads to sad attitude area improvement about death (17), the present research intended to study these subjects that “is spirituality dimensions development different in women for type II diabetes?” and“ are attitudes profiles related to death in women with type II diabetes different?”. Then relation between spirituality development and related attitudes on death would be discussed.

## Materials and Methods

In the percent research, cross-sectional design was used to review the association between spirituality and attitude among type II diabetic patients. Statistical population included women with type II diabetes in age group 18 to 78 years old who presented to outpatient center of Imam Khomeini Hospital, Sari, Iran from October 2011 to February 2012. One hundred patients were selected by convenience sampling method. Sample size was determined regarding previous studies (5). Inclusive criteria were type II diabetes recognition for at least one year during the use of oral anti-diabetic medications, and giving consent for participation in the research. Exclusive criteria were having other types of diabetes, taking insulin, present experience of intense problems concerning diabetes (by medical records), and presence of other chronic diseases (by patients’ report). Data were collected by the two tools explained as below: 

*Spiritual Assessment Inventory (SAI):* A self-report tool consisting 47 clauses that seven of which have two parts in which examinee declares his/her agreement or disagreement to each clauses in a five-point Likert scale. This questionnaire was designed by Hall and Edwards in 1996. It has two dimensions of ''awareness of God'' and "the quality of relationship with God.'' The questionnaire was revised in 2002 and the revised version includes 6 sub-scales (awareness, realistic acceptance, and disappointment, grandiosity, instability, and impression management). Hall and Edwards reported Cronbach's alpha coefficient of the questionnaire as follow: Awareness sub-scale = 0.95, realistic acceptance sub-scale = 0.90, disappointment sub-scale = 0.83, grandiosity sub-scale = 0.73, instability sub-scale = 0.84, impression management sub-scale = 0.77 (11). The Cronbach's alpha coefficient of the questionnaire has been reported to be 0.67 in Iranian population (18).

*Death Attitude Profile-Revised (DARP):* This questionnaire was designed by Wang Reker and Geser and consists of 32 clauses in a five-point Likert scale (from completely agree to completely disagree). The reported Cronbach's alpha coefficient of questionnaire was as follow: Death = 0.86, death avoidances = 0.88, neutral acceptance 0.65, approach acceptance = 0.97, escape acceptance = 0.84. Validity evaluation of the test has been reported as well by differential and convergent validity to other tests (19). The Cronbach's alpha coefficient of the questionnaire has been reported to be 0.63 to 0.87 in Iranian population (20).

The collected data were analyzed using SPSS for Windows 18.0 (SPSS inc., Chicago, IL, USA) and statistical tests F and Fisher's Least Significant Difference (LSD) tests and Pearson correlation coefficient were employed. To find differences in various death attitude aspects, first the equality of dependent variables’ co-variances was assessed by the Mauchly's test of sphericity. 

The probability level was rejected since the probability level of variance equality was less than 0.05 (W = 0.308, χ^2 ^= 9, p < 0.05). Thus, death attitude was analyzed using the Greenhouse-Geisser method.

## Results

Mean age of the sample was 48.11 years (range, 20-70 years). Regarding education, 50.6% had high school educations without high school diploma, 46.4% had high school diploma, 2% had bachelor’s degree and 1% had educational levels higher than bachelor’s degree. With respect to economic status, 13.0% had monthly income/earning less than IRR (Iranian Rial) 3,000,000; 42.0% between IRR 3,000,000 to IRR 6,000,000; 20.0% between IRR 6,000,000 to IRR 9,000,000; and 16.0% more than IRR 9,000,000. It should be mentioned that 9.0% of the subjects reported no information about their monthly income. About housing, 91.0% were home owner, 8.0% were leaseholder, and 1.0% used to living with others. 

Results of analyzing differences among various death attitude subscales are shown in table 1. Findings showed that the hypothesis based on the existence of differences in death attitude aspects was accepted. Fisher’s Least Significant Test (LSD) was used to study difference as post hoc tests (Table 2). Results showed this descending arrangement; neutral acceptance, approach acceptance, death fear, death avoidance and escape acceptance. All the dimensions were different except death avoidance and escape acceptance, which were classified in the same range.

To analyze the differences in spiritual development, the analyzing covariance matrix normality had to be dealt with. The probability level was rejected like Mauchly's test of sphericity to analyze covariance matrix equality because the probability level of variance equality was less than 0.05 (W = 0.053, χ^2^ = 14, p < 0.05). Thus, spiritual development was analyzed using the Greenhouse-Geisser method (Table 3). Fisher’s LSD was used to study difference as post hoc tests (Table 4). Results showed this descending arrangement; impression management, awareness of God dimension, realistic acceptance, grandiosity instability, and disappointment. This test indicated that "impression management" and "awareness of God" had the highest averages and "disappointment" had the lowest average.

**Table 1:** Analysis of the existence of differences among various death attitude subscales

**Source**	**SS** †	**df** ‡	**MS** §	**F** ||	**P-value**
**Death attitudes**	211.408	2.765	76.471	42.428	0.001
**Error**	488.308	270.925	1.802		

† Sum of Squares;

‡ Degrees of Freedom;

§ Mean Square;

|| Fisher

Thereafter, Pearson’s correlation coefficient was used to specify association between the spirituality dimensions and death attitude. Results are shown in table 5. Considering table 5 in confidence level 95% (CI = 95), “awareness of God” dimension showed positive association with approach acceptance profiles. “Realistic acceptance” was in positive association with fear of death, and in negative relation with “neutral acceptance”. Disappointment factor was in positive association with fear of death and “escape acceptance” profiles and was in negative correlation with “neutral acceptance”. Grandiosity factor was in positive correlation with “approach as acceptance” as well as “escape acceptance”. “Impression management” showed positive association with “approach acceptance” and was in negative correlation with “fear of death”. Instability demonstrated positive correlation with “death avoidance” and “escape acceptance”.

## Discussion

Low economic and educational status in the present sample was in accordance with results of Larranaga et al. based on this fact that there is more incidence of type II diabetes in groups with low socioeconomic status of society (3). Awareness of God dimension and impression management had the highest average in comparison with sub-scales of quality of relation to God. Classification of other factors concerning quality of relation to God in the present research showed this descending arrangement: instability, grandiosity, realistic acceptance and disappointment. Higher grades in impression management indicate exaggerative exposing of spiritual attitudes and behaviors that the most religious people do not experience them. It seems that higher grades in impression indicate lower spiritual health and people are not able to combine good and bad aspects of their ideas and experiences due to exaggerate spiritual attitudes and behaviors in confronting life facts. On the other hands, there is a possibility that these people image themselves better than other people, so they would more likely to be involved in ambitious illusions. Realistic acceptance that mentions person’s ability to combine positive experiences and disappointment in relation to God had a lower rank that may indicate quality of immature relation to God by person. It is probable that mystical thinking decrease spiritual health and it may prevent spiritual development and maturity and stop the person in lower level of spiritual development (11, 17).

**Table 2:** Mean, standard deviation (SD), and classification of death attitude subscales

**Source**	**Minimum**	**Maximum**	**Mean**	**SD**
**Fear of death**	1.29	6.86	4.535	1.283
**Avoidance of death**	1.00	7.00	4.132	1.707
**Neutral acceptance**	2.00	700	5.680	0.775
**Approach acceptance**	2.30	6.90	5.190	0.769
**Escape acceptance**	1.00	7.00	3.976	1.278

**Table 3:** Analysis of the existence of differences among various spiritual dimensions

**Source**	**SS** †	**df** ‡	**MS** §	**F** ||	**P-value**
**Spiritual Development**	390.102	2.025	192.606	29.977	0.001
**Error**	288.126	194.437	1.482		

† Sum of Squares;

‡ Degrees of Freedom;

§ = Mean Square;

|| = Fisher

**Table 4:** Mean, standard deviation (SD), and classification of spiritual dimensions

**Source**	**Minimum**	**Maximum**	**Mean**	**SD**
**Awareness of God**	2.63	4.95	4.073	0.475
**Realistic acceptance**	0.00	4.71	2.395	1.492
**Disappointment**	1.00	4.43	2.037	0.884
**Grandiosity**	1.43	5.00	2.378	0.797
**Impression management**	2.40	5.00	4.082	0.558
**Instability**	1.44	5.00	2.847	0.696

**Table 5:** Association between spiritual development and death attitude profiles

	Fear of death	Death avoidance	Neutral acceptance	Approach acceptance	Escape acceptance
Awareness of God	-0.174**	0.070 *	0.192	0.389 **	-0.020 **
Realistic Acceptance	0.271 **	0.169 *	-0.246 *	-0.104 **	0.189 **
Disappointment	0.203 *	0.137 *	-0.255 *	-0.096 **	0.313 **
Grandiosity	0.057 **	0.185 *	0.157	0.371 **	0.291 **
Impression Management	-0.212 *	-0.017 **	0.111	0.337 **	-0.006 **
Instability	0.121 **	0.206 *	-0.006 *	0.120 **	0.218 *

* p < 0.01;

** p < 0.05

Results of death fear profiles showed that natural death acceptance had the highest average followed by approach acceptance, death fear, escape acceptance, and death avoidance. In natural death acceptance profile, death is part of life process that is accepted as one of the life facts, while approach acceptance mentions person's tendency to die and giving award that is associated with belief in afterlife world. Existence of positive association between awareness of God with approach acceptance is in agreement with results of Dezuter et al. based on belief in afterlife world is associated with death acceptance approach (13, 21).

Association between instability in relation to God and profiles of attitudes to death showed that there is extreme halving for these people. These people have intense fear from death and they look to death as an attractive option to escape from life responsibilities such as what is seen in border personality disorder. Regarding high average of instability in the sample, considering this point needs more studies and investigations regarding probability of commit to suicide (6,12). Grandiosity in relation to God showed positive relation with profiles of approach acceptance and escape acceptance. It seems that ambition in addition to ambitious illusions increases person's hope to get excellent award (approach acceptance) or on the other hand, the person does not admit the life with his/her expects and tends to escape to abandon that life (escape acceptance). Moreover, studies showed that related obsession concerning death and anxiety is in positive relation with outer religious bias (14). Research results showed that there was positive association between realistic acceptance and death of fear, also negative correlation has been shown with neutral acceptance. This finding was in contrary to those studies that have shown death neutral acceptance is in positive association with mental health and fear of death is associated with more anxiety and depression (12, 19). So it is possible that realistic acceptance in relation to God in the present sample is in direction with good exposing about it and/or a defensive response, then person's disappointment experiences make incidence of death fear profile, on the other side, it is in negative relation with neutral profile.

Disappointment exists when person has unreasonable demands from God and not meeting them makes despair and disappointment. Disappointment has an important role in many psychological diseases (e.g., depression, anxiety, etc.). Results showed disappointment was in positive relation to fear of death and escape acceptance, also was in negative relation with neutral acceptance. It is probable that disappointment makes disorder in spiritual growth and due to its speculative rumination by it, escape profile has been appeared. It seems that belief on lack of award and punishment in afterlife world makes disappointment and anxiety as a result of that disappointment makes fear of death and death natural acceptance decrease. Research findings showed that awareness of the present sample of diabetic patients in relation to God is appropriate though quality of relation to God was immature and minor. According to the research record in the field of spirituality and its prohibitory role in incidence of mental disorders and its protective role in health and contradictory styles, this study showed that attention to spiritual development and maturity needs further studies. The samples were selected from individuals who were referred to public centers. Research on individuals referred to private centers also seems necessary. 

## Authors' contributions

This paper is written based on a thesis research (MA) of MN. MN designed the study, collected the clinical data, analyzed and wrote the manuscript. AK performed statistical analysis. YD helped in designing the study and writing the manuscript. All authors read and approved the final manuscript.

## Notes

**Declaration of interest:** None.
